# Colour scales with climate in North American ratsnakes: a test of the thermal melanism hypothesis using community science images

**DOI:** 10.1098/rsbl.2022.0403

**Published:** 2022-12-21

**Authors:** Maggie M. Hantak, Robert P. Guralnick, Alexander C. Cameron, Aaron H. Griffing, Sean M. Harrington, Jeffrey L. Weinell, Daniel J. Paluh

**Affiliations:** ^1^ Florida Museum of Natural History, University of Florida, Gainesville, FL 32611, USA; ^2^ Department of Biology and Museum of Southwestern Biology, University of New Mexico, Albuquerque, NM 87131, USA; ^3^ Department of Chemical and Biological Engineering, Princeton University, Princeton, NJ 08544, USA; ^4^ Department of Molecular Biology, Princeton University, Princeton, NJ 08544, USA; ^5^ Milwaukee Public Museum, Milwaukee, WI 53233, USA; ^6^ Department of Herpetology, American Museum of Natural History, New York, NY 10024-5192, USA; ^7^ INBRE Data Science Core, University of Wyoming, Laramie, WY 82071, USA; ^8^ Department of Ecology and Evolutionary Biology and Biodiversity Institute, University of Kansas, Lawrence, KS 66045, USA

**Keywords:** community science, iNaturalist, *Pantherophis*, thermal melanism hypothesis

## Abstract

Animal colour is a complex trait shaped by multiple selection pressures that can vary across geography. The thermal melanism hypothesis predicts that darker coloration is beneficial to animals in colder regions because it allows for more rapid solar absorption. Here, we use community science images of three closely related species of North American ratsnakes (genus *Pantherophis*) to examine if climate predicts colour variation across range-wide scales. We predicted that darker individuals are found in colder regions and higher elevations, in accordance with the thermal melanism hypothesis. Using an unprecedented dataset of over 8000 images, we found strong support for temperature as a key predictor of darker colour, supporting thermal melanism. We also found that elevation and precipitation are predictive of colour, but the direction and magnitude of these effects were more variable across species. Our study is the first to quantify colour variation in *Pantherophis* ratsnakes, highlighting the value of community science images for studying range-wide colour variation.

## 1. Introduction

Species colour and patterning is often the outcome of different selective pressures [1,2] and is used for concealment, communication and quality signalling, and physiological processes [3,4]. Colour also often varies geographically within and between species, which may relate to these multiple selection pressures operating locally. One key ecogeographic rule posited to explain variation in intraspecific colour patterning is the thermal melanism hypothesis [5,6], which predicts that darker animals will be found in colder climates as increased melanin aids in absorption of solar radiation [7]. Several studies on insects and reptiles have garnered support for this hypothesis [8–10], but the extent of this pattern across animals remains unknown.

North American ratsnakes (genus *Pantherophis*) are thought to vary in coloration across species' ranges. It has been long noted anecdotally by herpetologists that ratsnakes are particularly melanistic in northern populations, perhaps related to thermoregulatory benefits [11]. However, no studies have quantified intraspecific colour variation in this group or examined the relationship between colour and abiotic conditions. The North American ratsnake complex was historically considered a single species (*Pantherophis obsoletus*) with eight geographical subspecies distinguished by adult colour pattern [12]. The northern subspecies was defined by a dark grey/brown or black dorsal colour, while the remaining subspecies were characterized as various shades of grey, brown, yellow or orange ground colours (i.e. base) with blotches or stripes. However, molecular systematic work found that these colour-based subspecies do not represent distinct evolutionary lineages [13], and instead the complex is composed of four species [14]—*P. obsoletus* (distributed west of the Mississippi River), *P. spiloides* (distributed between the Mississippi River and Appalachian Mountains), *P. alleghaniensis* (distributed east of the Appalachian Mountains) and *P. bairdi* (in southwest Texas and northeastern Mexico)—all of which are highly variable in colour (electronic supplementary material, figure S1; see [15] for updated nomenclature).

Here, we test the importance of climate predictors on colour variation at the range-wide scale in eastern *Pantherophis* ratsnakes. We do so by using a novel approach that overcomes past limitations in examining snake coloration. Unfortunately, colour fades in fluid-preserved museum specimens, prohibiting use for such studies. In nature, snakes are difficult to find in high quantities, making it challenging for any one person or small team to assemble the needed data to study intraspecific patterns across a species range. Community science platforms, like iNaturalist, hold ever-growing image data resources of numerous species. These records are an invaluable resource for studying phenotypic variation across range-wide scales, especially for species that are not reliably discoverable (e.g. [16]). Di Cecco [17] highlight the utility of iNaturalist observations in biodiversity science, however, at present, the use of these images to examine species trait variation remains uncommon. We leverage over 8000 iNaturalist images to test range-wide colour and climate trends in *Pantherophis* ratsnakes. Based on the observed colour pattern cline in these species, we predicted that all species would conform to the thermal melanism hypothesis, with darker individuals found in colder climates and higher elevations (due to cooler temperatures and increased UV exposure) and lighter coloured snakes in warmer areas and lower elevations. In addition to climate-related variables, we also tested the alternative hypothesis of background matching as shown in other snakes [18,19], with the expectation that colour of eastern *Pantherophis* ratsnakes do not display this pattern.

## 2. Methods

### (a) Snake colour data

We downloaded all Research Grade images (years 1972–2020) of *P. alleghaniensis*, *P. spiloides* and *P. obsoletus* from iNaturalist (accessed 13 May 2020) using the R package *rinat* [20]. Research Grade records are georeferenced, and taxonomic identity is verified by at least two other iNaturalist users. The three *Pantherophis* species were selected based on similar latitudinal geographical distributions and variation in colour pattern. A total of 16 567 images were downloaded across species: 6731 *P. alleghaniensis*, 3335 *P. spiloides* and 6501 *P. obsoletus*.

To score *Pantherophis* colour pattern, we used the program ImageAnt (https://gitlab.com/stuckyb/imageant). Details on snake colour pattern scoring and record filtering can be found in the electronic supplementary material, Methods. Snake colour patterns were assigned into six categories: black, brown, dark grey, light grey, orange and yellow. Each image was initially scored by two independent co-authors. If there was incongruence between the first two scores, a third independent researcher provided a consensus.

We also tested for the possibility of background matching. We report the full methods in the electronic supplementary material for quantifying snake and background colours from images. In total, snake and background colour was quantified for 300 curated images of *P. alleghaniensis*, which was used as the test case because it varies the most in colour and latitudinal range.

### (b) Climate and geographical data

We downloaded the bioclimatic variables: annual mean temperature (BIO1), annual precipitation (BIO12), elevation and solar radiation at 30 arc-second (approx. 1 km) resolution (WorldClim V2.1; [21]). We had no expectation of seasonal variation in colour. Latitude was obtained from iNaturalist image metadata. Using the *raster* R package [22], we extracted the environmental variables for each record.

### (c) Statistical analyses

We used the *brms* package [23] to fit Bayesian generalized nonlinear regression models in R [24] with colour as a multinomial response and temperature, precipitation and elevation as predictors. Solar radiation and latitude were dropped from models because they were highly correlated with temperature (*r* > 0.9). Multi-collinearity was low among remaining co-variates (*r* < 0.40). Elevation was log transformed for normalization; all other predictors were normally distributed. Predictors were all mean-centered and standardized to compare effect sizes and for improved model performance. All models were fit with 2000 iterations across four MCMC chains and 1000 warm-up iterations. We ran four multinomial models, one full model including all three *Pantherophis* species with a random intercept of species and a separate model for each of the three species. Species-specific analyses were run due to evolutionary ambiguity in the group [15,25,26]. Black colour pattern was set as the comparative reference for all models; therefore, its parameter was fixed and other colour estimates are relative to it. For higher order models, we ran model selection using ‘loo_compare’ in the *brms* package and estimated model fit with the difference in their expected log predictive density using the leave-one-out cross-validation criterion (loo). For all models, we tested for interactions between temperature and precipitation, and while these models had a better fit, the interactions were weak and do not aid in the interpretation of the relationship between melanism and climate. We therefore focus on presenting in the main text models without interaction effects (see electronic supplementary material, table S1 and figure S2 for results with interactions included). In addition, we ran a reduced dataset in which we removed records with coordinate error greater than 1 km (see electronic supplementary material, Methods and Results). Lastly, we ran binary models to examine the influence of environment on a more generalized pattern of light or dark snake coloration (see electronic supplementary material, Methods and Results). We checked the residuals of the binary model with species as a fixed effect and found no evidence of spatial autocorrelation (electronic supplementary material, figure S3). To examine the possibility of background matching, we ran a linear regression with quantified snake colour as the response and mean-centered and standardized background colour and latitude (as an alternative predictor) as the predictors.

## 3. Results

**Figure 1.**
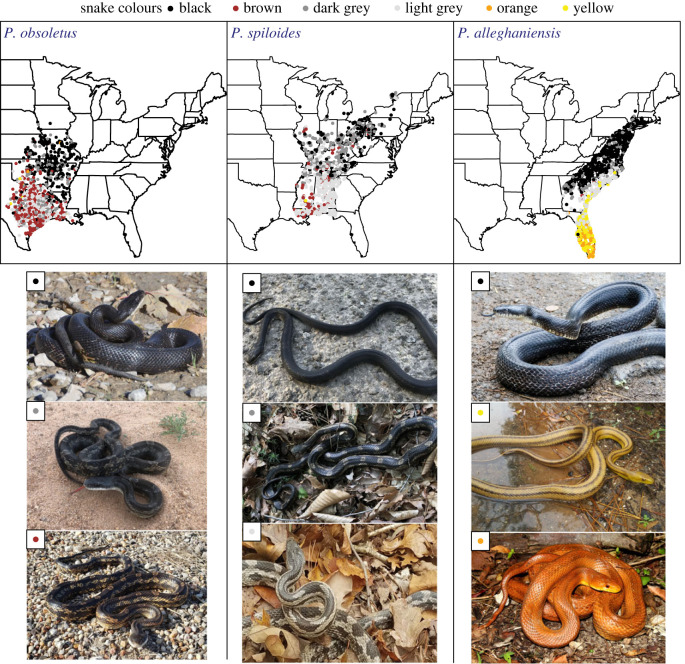
Maps of the range-wide distribution of *Pantherophis obsoletus, P. spiloides* and *P. alleghaniensis* based on iNaturalist records clipped to each species IUCN distribution. Records are coloured by scored snake colour pattern (black, brown, dark grey, light grey, orange or yellow) for each species. Images below each map display colour pattern variation observed within each respective species. Images credit top to bottom by species: *P. obsoletus*—Chrissy McClarren and Andy Reago (no. 163675470), David Kelley (no. 130911586), Cristy Wade (no. 171898208); *P. spiloides*—Robb Hannawacker (no. 8944885), John Kees (no. 64287651), Anne (no. 64478146); *P. alleghaniensis*—Mirko Schoenitz (no. 8516891), Daniel Estabrooks (no. 19861331), J.D. Willson (no. 59436623).

**Figure 2.**
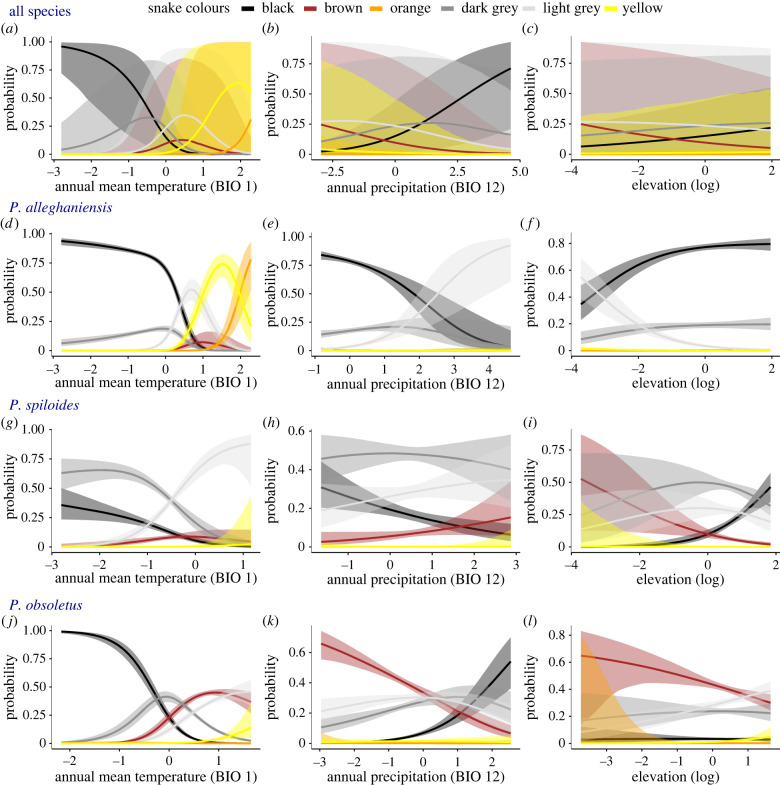
Conditional effects plots with 95% credible intervals demonstrating the effects of annual mean temperature (BIO 1), annual precipitation (BIO 12) and elevation in determining the probability of observing a particular colour variant for all snakes (species combined; (*a*–*c*)); *Pantherophis alleghaniensis* (*d*–*f*); *P. spiloides* (*g*–*i*) and *P. obsoletus* (*j*–*l*).

Out of 16 567 initial iNaturalist images, we obtained consensus scores for 8279 adult *Pantherophis* records. After further filtering, we generated a final dataset of 8098 records (3220 *P. alleghaniensis*, 1703 *P. spiloides*, 3175 *P. obsoletus;* figure 1; electronic supplementary material, figure S1 and table S2). For each analysis of colour variation (all species and separate), model selection indicated that the best-fit models included all predictor variables (electronic supplementary material, table S3). In the full model, with species as a random effect, the probability of black snake occurrence was highest in colder areas and decreased with warming temperature (figure 2*a* and table 1). Temperature was the strongest effect, and probability of presence across temperature gradients differed the most between black snakes compared to orange and yellow snakes (figure 2*a*). Probability of black snake presence increased with increasing precipitation (figure 2*b*) and elevation (figure 2*c*), but elevation was not an important predictor separating black snake occurrence from yellow and orange snake occurrence (table 1).

**Table 1.** Fixed-effect parameter estimates for the best-fit model of colour variation across all snake species. Black colour pattern was set as the comparative reference for all models. Estimates in the table represent mean probability differences of snake colour patterns compared to black snakes for each predictor variable (temperature, precipitation and elevation). Italicized denotes significant effects where the 95% Bayesian credible interval (CI) does not overlap zero.

variable	colour comparison	estimate	estimate error	lower 95% CI	upper 95% CI
intercept	black–brown	−0.54	1.71	−3.92	2.77
	black–dark grey	0.34	1.16	−2.01	2.64
	black–light grey	0.32	1.52	−2.66	3.43
	*black–orange*	*−9.96*	*3.37*	*−14.64*	*−1.12*
	black–yellow	−2.35	1.79	−5.69	1.38
temperature	*black–brown*	*3.93*	*0.13*	*3.67*	*4.18*
	*black–dark grey*	*1.26*	*0.07*	*1.13*	*1.39*
	*black–light grey*	*4.10*	*0.11*	*3.89*	*4.32*
	*black–orange*	*10.21*	*0.53*	*9.23*	*11.31*
	*black–yellow*	*6.85*	*0.22*	*6.43*	*7.29*
precipitation	*black–brown*	*−0.92*	*0.08*	*−1.07*	*−0.76*
	*black–dark grey*	*−0.39*	*0.06*	*−0.50*	*−0.28*
	*black–light grey*	*−0.67*	*0.07*	*−0.80*	*−0.53*
	*black–orange*	*−1.01*	*0.34*	*−1.68*	*−0.34*
	*black–yellow*	*−0.98*	*0.17*	*−1.30*	*−0.67*
elevation	*black–brown*	*−0.46*	*0.09*	*−0.63*	*−0.29*
	*black–dark grey*	*−0.13*	*0.05*	*−0.22*	*−0.03*
	*black–light grey*	*−0.25*	*0.06*	*−0.38*	*−0.13*
	black–orange	0.02	0.26	−0.48	0.53
	black–yellow	−0.02	0.12	−0.25	0.21

In the single species models, all three species showed strong, significant trends of higher probability of black snake presence in colder environments (figure 2*d,g*,*j*). The main differences were in other climate factors, especially precipitation. *Pantherophis obsoletus* showed a significantly higher probability of black snake presence in wetter areas compared to other colour forms (figure 2*k*), but the other two species showed weaker or a non-significant relationship in the opposite direction (e.g. higher probability of black snakes in drier locations; figure 2*e*,*h*). Elevation was not an important predictor separating black snake occurrence from other dark forms for *P. alleghaniensis* (but was predictive for lighter forms) and the effects are even weaker for *P. obsoletus* (table 2). However, elevation was a strong effect for *P. spiloides*, where black forms always have a higher probability of occurrence at higher elevations compared to other colour forms (figure 2*i*).

**Table 2.** Fixed-effect parameter estimates for the best-fit model of colour variation separated by species: *Pantherophis alleghaniensis*, *P. spiloides* and *P. obsoletus*. Black colour pattern is the comparative reference for all models. Estimates in the table represent mean probability differences of snake colour patterns compared to black snakes for each predictor variable (temperature, precipitation and elevation). Italicized denotes significant effects where the 95% Bayesian credible interval (CI) does not overlap zero.

variable	colour comparison	*P. alleghaniensis*				*P. spiloides*				*P. obsoletus*			
		estimate	estimate error	lower 95% CI	upper 95% CI	estimate	estimate error	lower 95% CI	upper 95% CI	estimate	estimate error	lower 95% CI	upper 95% CI
intercept	black–brown	*−5.13*	*0.62*	*−6.43*	*−4.00*	0.76	0.42	−0.04	1.56	*−0.95*	*0.14*	*−1.22*	*−0.69*
	black–dark grey	*−1.42*	*0.09*	*−1.61*	*−1.25*	*1.60*	*0.25*	*1.12*	*2.09*	−0.04	0.10	−0.23	0.15
	black–light grey	*−2.78*	*0.18*	*−3.14*	*−2.44*	*2.62*	*0.31*	*2.01*	*3.23*	*−1.44*	*0.16*	*−1.77*	*−1.12*
	black–orange	*−14.23*	*0.97*	*−16.17*	*−12.37*					*−5.51*	*0.87*	*−7.45*	*−4.07*
	black–yellow	*−5.78*	*0.43*	*−6.67*	*−5.00*	−2.18	1.27	−4.93	0.09	*−5.70*	*0.59*	*−6.91*	*−4.59*
temperature	black–brown	*6.22*	*0.67*	*4.99*	*7.59*	*1.69*	*0.42*	*0.90*	*2.51*	*4.57*	*0.24*	*4.12*	*5.05*
	black–dark grey	*0.50*	*0.11*	*0.29*	*0.71*	0.27	0.15	−0.02	0.57	*2.53*	*0.18*	*2.20*	*2.89*
	black–light grey	*4.68*	*0.33*	*4.07*	*5.33*	*2.50*	*0.33*	*1.88*	*3.14*	*5.08*	*0.26*	*4.57*	*5.59*
	black–orange	*12.45*	*0.74*	*11.03*	*13.89*					*1.83*	*0.97*	*0.04*	*3.78*
	black–yellow	*8.08*	*0.42*	*7.28*	*8.90*	*3.44*	*1.33*	*0.73*	*6.03*	*6.88*	*0.72*	*5.51*	*8.29*
precipitation	black–brown	−0.29	0.95	−2.24	1.44	*0.74*	*0.27*	*0.20*	*1.27*	*−1.45*	*0.15*	*−1.74*	*−1.16*
	black–dark grey	*0.33*	*0.12*	*0.10*	*0.55*	*0.32*	*0.13*	*0.08*	*0.57*	*−0.90*	*0.13*	*−1.15*	*−0.65*
	black–light grey	*1.39*	*0.25*	*0.91*	*1.88*	*0.49*	*0.20*	*0.09*	*0.88*	*−1.11*	*0.15*	*−1.39*	*−0.80*
	black–orange	0.39	0.55	−0.66	1.47					−1.46	0.86	−3.25	0.12
	black–yellow	0.76	0.44	−0.09	1.61	0.68	0.81	−0.85	2.35	*−1.05*	*0.33*	*−1.70*	*−0.41*
elevation	black–brown	−0.12	0.42	−0.91	0.75	*−1.71*	*0.32*	*−2.32*	*−1.09*	−0.13	0.22	−0.55	0.29
	black–dark grey	0.00	0.07	−0.13	0.13	*−1.08*	*0.19*	*−1.45*	*−0.70*	0.06	0.19	−0.32	0.43
	black–light grey	*−0.96*	*0.10*	*−1.16*	*−0.76*	*−1.08*	*0.29*	*−1.64*	*−0.51*	0.29	0.22	−0.13	0.72
	black–orange	*−0.80*	*0.32*	*−1.43*	*−0.15*					0.03	1.21	−2.15	2.60
	black–yellow	*−0.95*	*0.17*	*−1.29*	*−0.63*	*−1.44*	*0.63*	*−2.64*	*−0.14*	*1.05*	*0.43*	*0.23*	*1.89*

We found no evidence of background matching in *Pantherophis*. Quantified background colour was not explanatory in the linear model (*β* = 0.93, s.e. = 0.83, *p* = 0.258), but latitude was important (*β* = −9.21, s.e. = 0.83, *p* < 0.001).

## 4. Discussion

### (a) Climate-related drivers of colour variation

Several studies on snake colour variation support the thermal melanism hypothesis [27–29]. This hypothesis suggests that melanistic individuals have an advantage in cooler temperatures because darker colour allows for faster heat absorption and increased overall body temperatures compared to lighter individuals [7,30]. Our results also demonstrate support for thermal melanism influencing the distribution of colour forms in *Pantherophis* ratsnakes. Using mixed modelling and individually modelling each species of *Pantherophis*, we found that temperature was the most important predictor separating black colour forms from lighter coloured snakes. Previous studies on North American ratsnakes have hypothesized that colour scales with climate along temperature gradients [11,13]; however, our study is the first to test this prediction. Further, our approach of testing climate drivers across multiple closely related species with similar latitudinal ranges provides a critical means to determine consistency across species.

Across all *Pantherophis*, melanistic colour forms were more common in areas that experience higher precipitation. Individual species responses demonstrate that *P. obsoletus* follows this general trend, whereas melanistic forms of *P. alleghaniensis* and *P. spiloides* are less likely to occur in areas with higher precipitation. One ecogeographic pattern to explain this trend in *P. obsoletus* is Gloger's rule, which predicts that darker coloured animals are favoured in warm, wet environments [31]. There are multiple working hypotheses to explain the pattern of Gloger's rule, including increased camouflage, protection from solar radiation and/or parasites, or pleiotropic effects [31,32]. However, studies that more carefully control for joint effects of temperature and precipitation often demonstrate that darker coloured individuals are more likely found in colder areas (i.e. thermal melanism), and areas with higher precipitation [33–35]. Given this, recent work suggests redefining Gloger's rule to be based on only precipitation and/or humidity and not temperature [33,34]. Still, across these three closely related species found in close proximity, a more narrowly defined Gloger's rule is the exception rather than the rule. While we frame our results here through the lens of thermal ecology and landscape-level patterns, with a focus on comparing more melanistic colour forms to lighter ones, this is likely oversimplistic [36] and a suite of non-climate-related factors, such as geographical barriers, biotic interactions or neutral genetic processes [37] may also be important in determining spatial patterns of intraspecific colour variation. Further work especially examining the distribution of lighter individuals (e.g. orange and yellow forms) and accounting for these non-climatic factors, may help uncover complex interactions and spatially varying selection pressures.

Finally, we expected relatively cooler temperatures and increased UV exposure at higher elevations, and therefore, predicted snakes would be darker in those areas. Across all species, the presence of black snakes increased in higher elevations. This trend appears to be driven by the distribution of *P. spiloides* colour forms, which was more strongly affected by elevation. An examination of elevational differences between the three *Pantherophis* species reveals that *P. spiloides* is generally found at higher elevations than *P. alleghaniensis* or *P. obsoletus*, suggesting factors along elevation gradients may exert stronger selection pressures for this species (electronic supplementary material, figure S4). In *P. alleghaniensis*, elevation is important for separating black snakes from lighter coloured individuals, likely driven by the high occurrence of lighter coloured snakes in the southeastern portion of the U.S., e.g. along the Atlantic Coastal Plain, which, in general, is low in elevation [38]. It is therefore also possible that the correlation between colour and elevation is a byproduct of the evolutionary history of this species [25].

While our study finds strong evidence of environmental variables influencing the distribution *Pantherophis* ratsnake coloration, other mechanisms, such as camouflage may also play a role in colour distribution. We found no evidence of background matching in *P. alleghaniensis*, but further examination with data collected from standardized images would be useful in testing the possibility of crypsis in this group. Finally, we note that snake dorsal patterning (striped or blotched) may also contribute to intraspecific variation in heat absorption, but examining this trait was not within the scope of our study. Further work quantifying snake pattern variation across spatial and environmental gradients would help extend the work presented here.

### (b) Evolutionary patterns of colour variation

Increased melanin in northern populations of *P. obsoletus, P. alleghaniensis* and *P. spiloides* may have evolved several times independently [13]. *Pantherophis obsoletus* is more closely related to *P. bairdi*—a non-melanistic ratsnake species (ground colour is either pale orange, yellow or red with stripes) that has a limited distribution in southwest Texas and northeastern Mexico—than to the clade containing *P. alleghaniensis* and *P. spiloides*. Thus, it may be that the colour pattern of the most recent common ancestor of the North American ratsnake complex was non-melanistic [13] because all *Pantherophis* ratsnake species share a non-melanistic colour pattern as juveniles (light grey and blotched) and other *Pantherophis* species (foxsnakes and corn snakes) also lack dark pigmentation. Recent work has shown that significant introgression has persisted between the four species of *Pantherophis* ratsnakes where they come into contact [25], with some arguing that these still comprise a single structured species [24]. Thus, it is also possible that alleles coding for black coloration arose once and have introgressed across species and population boundaries. Investigating whether the molecular mechanism of increased melanin is shared in the northern populations of *P. obsoletus, P. alleghaniensis* and *P. spiloides* through comparative genomics and transcriptomics is a key next step in understanding the evolution and distribution of coloration in this group. Although our work is largely focused on melanism, in warmer parts of the species ranges, snake colour can be orange or yellow. The highest occurrence of orange and yellow colour is in *P. alleghaniensis* throughout the state of Florida (figure 1). Divergence of *P. alleghaniensis* may have occurred in Florida due to interglacial sea-level changes [25]. Thus, yellow coloration may also be the ancestral condition of this group, but the adaptive function of the yellow and orange colours remains unclear.

### (c) Community science for examining species colour variation

Exploring broad-scale questions of species phenotypic patterns has become more tractable with the ever-growing resource of community science images [17]. Using Google Images, Leighton *et al*. [39] spearheaded the use of community-sourced images to study trait variation. More recently, studies have harnessed the abundance of iNaturalist community science images to examine intraspecific variation in traits like coloration [40–43], in addition to studying phenological trends [44–47] and predation rates [16]. Our study is the first to test range-wide colour and climate associations in North American ratsnakes, which is likely due to the challenges with documenting colour in these snakes. Our methodology of manually scoring snake coloration from iNaturalist images worked well for our study; however, streamlined high-throughput techniques are necessary to keep pace with the continuous growth of image resources. For instance, we downloaded 16 567 ratsnake images on 13 May 2020, and as of 17 March 2022 records for the three species examined more than doubled to 34 151 images. Leveraging artificial intelligence approaches, such as deep learning, is one such method for keeping pace with the rapid growth of community science images. For example, de Solan *et al*. [48] used deep learning to quantify snake mimicry from standardized digital photographs. However, image heterogeneity is inherent in community science images, and Hantak *et al*. [42] developed a highly accurate deep learning model to score a salamander colour polymorphism from iNaturalist images. That study was based on a simple binary classifier (stripe presence/absence), while ratsnake colour categorization is more challenging as colour variation may be more continuous rather than discrete. Photographs taken from a variety of cameras under diverse lighting conditions, with no calibration, highlights the requirement of our repeatable colour-binning approach. Important next steps will be to simultaneously explore the possibility for machine-based colour classifications and better testing of how to derive meaningful continuous colour information from digital community science images, despite challenges with image heterogeneity [49,50].

## Data Availability

Data and code for this study are available in the Zenodo repository: https://doi.org/10.5281/zenodo.7190697 [51]. The data are provided in the electronic supplementary material [52].

## References

[1] Hingston RWG. 1933 The meaning of animal colour and adornment. London, UK: Edward Arnold.

[2] Cott HB. 1940 Adaptive coloration in animals. London, UK: Methuen & Co. Ltd.

[3] Cuthill IC et al. 2017 The biology of color. Science **357**, eaan0221. (10.1126/science.aan0221) 28774901

[4] Endler JA, Mappes J. 2017 The current and future state of animal coloration research. Phil. Trans. R. Soc. B **372**, 20160352. (10.1098/rstb.2016.0352) 28533467 PMC5444071

[5] Watt WB. 1968 Adaptive significance of pigment polymorphisms in *Colias* butterflies. I. Variation of melanin pigment in relation to thermoregulation. Evolution **22**, 437-458. (10.2307/2406873) 28564757

[6] Gaston KJ, Chown SL, Evans KL. 2008 Ecogeographical rules: elements of a synthesis. J. Biogeogr. **35**, 483-500. (10.1111/j.1365-2699.2007.01772.x)

[7] Clusella-Trullas S, van Wyk JH, Spotila JR. 2007 Thermal melanism in ectotherms. J. Therm. Biol. **32**, 235-245. (10.1016/j.jtherbio.2007.01.013)

[8] True JR. 2003 Insect melanism: the molecules matter. Trends Ecol. Evol. **18**, 640-647. (10.1016/j.tree.2003.09.006)

[9] Clusella-Trullas S, Terblanche JS, Blackburn TM, Chown SL. 2008 Testing the thermal melanism hypothesis: a macrophysiological approach. Funct. Ecol. **22**, 232-238. (10.1111/j.1365-2435.2007.01377.x)

[10] Clusella-Trullas S, van Wyk JH, Spotila JR. 2009 Thermal benefits of melanism in cordylid lizards: a theoretical and field test. Ecology **90**, 2297-2312. (10.1890/08-1502.1) 19739391

[11] Braswell AL. 1977 Geographic variation in *Elaphe obsoleta* (Say) (Reptilia, Squamata, Colubridae) in North Carolina. PhD thesis, North Carolina State University, Raleigh, NC.

[12] Conant R, Collins JT. 1991 A field guide to reptiles and amphibians: eastern and central North America, 3rd edn. Boston, MA: Houghton Mifflin.

[13] Burbrink FT, Lawson R, Slowinski JB. 2000 Mitochondrial DNA phylogeography of the polytypic North American rat snake (*Elaphe obsoleta*): a critique of the subspecies concept. Evolution **54**, 2107-2118. (10.1111/j.0014-3820.2000.tb01253.x) 11209786

[14] Burbrink FT. 2001 Systematics of the eastern ratsnake complex (*Elaphe obsoleta*). Herpetol. Monogr. **15**, 1-53. (10.2307/1467037)

[15] Burbrink FT, Pyron RA, Gehara MC, McKelvy AD, Myers EA. 2021 The corrected taxonomic history of the North American ratsnakes (*Pantherophis obsoletus* complex). Herpetol. Rev. **53**, 537-547.

[16] Putman BJ, Williams R, Li E, Pauly GB. 2021 The power of community science to quantify ecological interactions in cities. Sci. Rep. **11**, 3069. (10.1038/s41598-021-82491-y) 33542360 PMC7862361

[17] DiCecco GJ, Barve V, Belitz MW, Stucky BJ, Guralnick RP, Hurlbert AH. 2021 Observing the observers: how participants contribute data to iNaturalist and implications for biodiversity science. Bioscience **71**, 1179-1188. (10.1093/biosci/biab093)

[18] Isaac LA, Gregory PT. 2013 Can snakes hide in plain view? Chromatic and achromatic crypsis of two colour forms of the western terrestrial garter snake (*Thamnophis elegans*). Biol. J. Linn. Soc. **108**, 756-772. (10.1111/bij.12020)

[19] Wilson D, Heinsohn R, Endler JA. 2007 The adaptive significance of ontogenetic colour change in a tropical python. Biol. Lett. **3**, 40-43. (10.1098/rsbl.2006.0574) 17443961 PMC2373822

[20] Barve V, Hart E 2022 rinat: Access ‘iNaturalist’ data through APIs. See https://cran.r-project.org/web/packages/rinat/index.html.

[21] Fick SE, Hijmans RJ. 2017 WorldClim 2: new 1-km spatial resolution climate surfaces for global land areas. Int. J. Climatol. **37**, 4302-4315. (10.1002/joc.5086)

[22] Hijmans RJ 2020 *raster: geographic data analysis and modeling*. R Package. See https://cran.r-project.org/web/packages/raster/raster.pdf.

[23] Bürkner PC. 2017 brms: an R package for Bayesian multilevel models using Stan. J. Stat. Softw. **80**, 1-28. (10.18637/jss.v080.i01)

[24] R Core Team 2021 R: a language and environment for statistical computing. Vienna, Austria: R Foundation for Statistical Computing. See https://www.R-project.org/.

[25] Burbrink FT, Gehara M, McKelvy AD, Myers EA. 2021 Resolving spatial complexities of hybridization in the context of the gray zone of speciation in North American ratsnakes (*Pantherophis obsoletus* complex). Evolution **75**, 260-277. (10.1111/evo.14141) 33346918

[26] Hillis DM, Wüster W. 2021 Taxonomy and nomenclature of the *Pantherophis obsoletus* complex. Herpetol. Rev. **52**, 51-52.

[27] Gibson AR, Falls B. 1979 Thermal biology of the common garter snake *Thamnophis sirtalis* L. II. The effects of melanism. Oecologia **43**, 99-109. (10.1007/BF00346675) 28309830

[28] Bittner TD, King RB, Kerfin MJ. 2002 Effects of body size and melanism on the thermal biology of garter snakes (*Thamnophis sirtalis*). Copeia **2002**, 477-482. (10.1643/0045-8511(2002)002[0477:EOBSAM]2.0.CO;2)

[29] Martínez-Freiría F, Toyama KS, Freitas I, Kaliontzopoulou A. 2020 Thermal melanism explains macroevolutionary variation of dorsal pigmentation in Eurasian vipers. Sci. Rep. **10**, 16122. (10.1038/s41598-020-72871-1) 32999337 PMC7528074

[30] Forsman A. 1995 Heating rates and body temperature variation in melanistic and zigzag *Vipera berus*: does colour make a difference? Ann. Zool. Fennici **32**, 365-374.

[31] Delhey K. 2017 Gloger's rule. Curr. Biol. **27**, R689-R691. (10.1016/j.cub.2017.04.031) 28743010

[32] Cerezer FO, Ribeiro JR, Graipel M, Cáceres NC. 2020 The dark side of coloration: ecogeographical evidence supports Gloger's rule in American marsupials. Evolution **74**, 2046-2058. (10.1111/evo.13989) 32395852

[33] Delhey K. 2019 A review of Gloger's rule, an ecogeographical rule of colour: definitions, interpretations and evidence. Biol. Rev. **94**, 1294-1316. (10.1111/brv.12503) 30892802

[34] Delhey K, Dale J, Valcu M, Kempenaers B. 2019 Reconciling ecogeographical rules: rainfall and temperature predict global col our variation in the largest bird radiation. Ecol. Lett. **22**, 726-736. (10.1111/ele.13233) 30779293

[35] Marcondes RS, Nations JA, Seeholzer GF, Brumfield RT. 2021 Rethinking Gloger's rule: climate, light environments and color in a large family of tropical birds (Furnariidae). Am. Nat. **197**, 592-606. (10.1086/713386) 33908827

[36] Riemer K, Guralnick RP, White EP. 2018 No general relationship between mass and temperature in endothermic species. eLife **7**, e27166. (10.7554/eLife.27166) 29313491 PMC5760208

[37] Machado AP, Cumer T, Iseli C, Beaudoing E, Dupasquier M, Guex N, Goudet J. 2021 Unexpected post-glacial colonisation route explains the white colour of barn owls (*Tyto alba*) from the British Isles. Mol. Ecol. **31**, 482-497. (10.1111/mec.16250) 34695244 PMC9298239

[38] Kosovich JJ. 2008 State of Florida 1:24,000- and 1:100,000-scale quadrangle index map—highlighting low-lying areas derived from USGS digital elevation models: U.S. Geological Survey Scientific Investigations Map 3047, scale 1:1,000,000. See https://pubs.usgs.gov/sim/3047/downloads/SIM3047.pdf.

[39] Leighton GRM, Hugo PS, Roulin A, Amar A. 2016 Just Google it: assessing the use of Google Images to describe geographical variation in visible traits of organisms. Methods Ecol. Evol. **7**, 1060-1070. (10.1111/2041-210X.12562)

[40] Lehtinen RM, Carlson BM, Hamm AR, Riley AG, Mullin MM, Gray WJ. 2020 Dispatches from the neighborhood watch: using citizen science and field survey data to document color morph frequency in space and time. Ecol. Evol. **10**, 1526. (10.1002/ece3.6006) 32076531 PMC7029058

[41] Cosentino BL, Gibbs JP. 2022 Parallel evolution of urban–rural clines in melanism in a widespread mammal. Sci. Rep. **12**, 1752. (10.1038/s41598-022-05746-2) 35110609 PMC8810909

[42] Hantak MM, Guralnick RP, Zare A, Stucky BJ. 2022 Computer vision for assessing species color pattern variation from web-based community science images. *iScience* **25**, 104784. (10.1016/j.isci.2022.104784)

[43] Lattanzio MS, Buontempo MJ. 2021 Ecogeographic divergence linked to dorsal coloration in eastern hog-nosed snakes (*Heterodon platirhinos*). Herpetologica **77**, 134-145. (10.1655/Herpetologica-D-19-00031.1)

[44] Barve VV et al. 2020 Methods for broad-scale plant phenology assessments using citizen scientists’ photographs. Appl. Plant Sci. **8**, e11315. (10.1002/aps3.11315) 31993257 PMC6976896

[45] Belitz MW et al. 2021 Climate drivers of adult insect activity are conditioned by life history traits. Ecol. Lett. **24**, 2687-2699. (10.1111/ele.13889) 34636143

[46] Brenskelle L, Barve V, Majure LC, Guralnick RP, Li D. 2021 Analyzing phenological anomaly in *Yucca* of the southwestern United States. Sci. Rep. **11**, 20819. (10.1038/s41598-021-00265-y) 34675272 PMC8531367

[47] Li D et al. 2021 Climate, urbanization, and species traits interactively drive flowering duration. Glob. Change Biol. **27**, 892-903. (10.1111/gcb.15461) 33249694

[48] de Solan T, Renoult JP, Geniez P, David P, Crochet PA. 2020 Looking for mimicry in a snake assemblage using deep learning. Am. Nat. **196**, 74-86. (10.1086/708763) 32552103

[49] Laitly A, Callaghan CT, Delhey K, Cornwell WK. 2021 Is color data from citizen science photographs reliable for biodiversity research? Ecol. Evol. **11**, 4071-4083. (10.1002/ece3.7307) 33976795 PMC8093748

[50] Lürig M, Donoughe S, Svensson EI, Porto A, Tsuboi M. 2021 Computer vision, machine learning, and the promise of phenomics in ecology and evolutionary biology. Front. Ecol. Evol. **9**, 642774. (10.3389/fevo.2021.642774)

[51] Hantak MM, Guralnick RP, Cameron AC, Griffing AH, Harrington SM, Weinell JL, Paluh DJ. 2022 Data from: Colour scales with climate in North American ratsnakes: a test of the thermal melanism hypothesis using community science images. Zenodo. (10.5281/zenodo.7190697) PMC9768630 36541094

[52] Hantak MM, Guralnick RP, Cameron AC, Griffing AH, Harrington SM, Weinell JL, Paluh DJ. 2022 Colour scales with climate in North American ratsnakes: a test of the thermal melanism hypothesis using community science images. *Figshare*. (10.6084/m9.figshare.c.6340208) PMC9768630 36541094

